# A bibliometric and visualized analysis of heartland virus

**DOI:** 10.3389/fmicb.2024.1509749

**Published:** 2025-01-13

**Authors:** Huiying Zhang, Leiliang Zhang

**Affiliations:** ^1^Department of Clinical Laboratory Medicine, The First Affiliated Hospital of Shandong First Medical University and Shandong Provincial Qianfoshan Hospital, Jinan, China; ^2^Department of Pathogen Biology, School of Clinical and Basic Medical Sciences, Shandong First Medical University and Shandong Academy of Medical Sciences, Jinan, China

**Keywords:** heartland virus, HRTV, bibliometric, CiteSpace, VOSviewer

## Abstract

**Background:**

Heartland virus (HRTV) is an emerging tick-borne bunyavirus first detected in 2009. The purpose of this study was to utilize bibliometric analysis to assess the research trends, key foci, and progress of HRTV. This analysis aims to provide valuable references and insights for future basic research and prevention and control of HRTV to promote the progress and development of related fields.

**Methods:**

The Web of Science Core Collection (WOSCC) was used to extract global publications on the HRTV from 2013 to 2024. VOSviewer, CiteSpace, Scimago Graphica, and Bibliometrix were used to process the data and visualize the results.

**Results:**

A stable trend in publication numbers was observed, with 82 articles from 17 countries. The United States led in publications, with significant contributions from the Centers for Disease Control and Prevention-USA. Keywords indicated research emphasis on “Heartland virus” and “severe fever.”

**Conclusion:**

HRTV research is in a phase of continuous and progressive growth, with a steady literature output over the past decade, indicating this field’s wide interest and importance in the research community. Currently, researchers are focusing on pathogenesis, immune response, vector relationships, and epidemiology, providing valuable insights for future studies.

## 1 Introduction

Heartland virus (HRTV), an emerging bunyavirus, was first detected in Missouri in 2009 and received further reports and attention in 2012 (Esguerra, 2016) The pathogenicity of the virus is on the rise in the United States. A retrospective study reported that vertebrates carrying neutralizing antibodies specific to HRTV had been detected in states where no human infections have been documented, a finding that suggests that the geographic distribution of HRTV may be expanding beyond the known areas of case occurrence, demonstrating a high degree of pathogenicity (Feng et al., 2024) HRTV has significant gene sequence similarity to severe fever with thrombocytopenia syndrome virus (SFTSV), a tick-borne bunyavirus transmitted through the tick bite of the *Haemaphysalis longicornis* and potentially other tick species, which produces similar symptoms in China, Korea, and Japan (Brault et al., 2018; Dembek et al., 2024) HRTV is classified in the genus *Bandavirus* in the virus classification, belonging to the family *Phenuiviridae* and the order Bunyavirales, and is represented as the second most important species of *Bandavirus* after SFTSV (Kuhn et al., 2022) Symptoms of HRTV infection are varied and usually include fever, lethargy, marked fatigue, headache, muscle aches, loss of appetite, nausea, diarrhea, weight loss, joint pain, decreased white blood cell counts, and decreased platelet counts. In some cases, these symptoms may worsen further, triggering more serious consequences such as bleeding disorders, multiple organ failure, or even life-threatening conditions (Dembek et al., 2024) We need an in-depth understanding of the specific mechanisms of HRTV replication, how the virus interacts with its host, the mechanisms of viral pathogenesis, and the degree of virulence (Feng et al., 2024) There are currently no available vaccines or approved treatment regimens for the prevention and treatment of HRTV disease, indicating the need for further detailed research (Westover et al., 2024).

The goal of this study is to utilize bibliometric methods to provide an in-depth analysis of the literature in the field of HRTV to provide a clearer picture of the current status and future trends of HRTV-based research. In addition, a systematic review and assessment of HRTV-related publications published in the Web of Science Core Collection (WOSCC) will be conducted. We used the analysis software VOSviewer and CiteSpace to conduct an exhaustive bibliometric analysis of HRTV-related literature published since 2013. Visual knowledge maps were developed in the process, which covers several key dimensions such as the number of publications, countries, research organizations, major publishing journals, prolific authors, and keyword co-occurrence analyses, thus providing us with a comprehensive overview of research in HRTV. By conducting a bibliometric analysis of HRTV, researchers can discern prominent research trends and pressing issues in this domain, offering significant guidance for research endeavors and preventive measures. Furthermore, this methodology aids in evaluating the influence of research papers, authors, and journals on HRTV, achieving a more objective measurement of the importance and impact of HRTV research through metrics such as the number of citations, citation frequency, and other indicators. This, in turn, provides robust support for academic evaluations and research funding applications. Additionally, an analysis of collaborative relationships within the literature sheds light on the networks and patterns of cooperation among nations, authors, and institutions, fostering the establishment of suitable research partnerships, the discovery of potential collaborators, and the promotion of academic exchanges. Analyzing the institutions and journals that publish the most pertinent papers in the field of HRTV offers researchers valuable guidance in selecting collaborative institutions and suitable outlets for their research publications. Ultimately, tracking temporal changes in the literature reveals the evolution of knowledge and advancements in HRTV research, inspiring researchers to innovate. By revealing the developmental lineage of HRTV, current research focuses, and cutting-edge developments, this study aims to provide unique insights and valuable references for future HRTV research and prevention efforts to help promote the continued progress and development of the field.

## 2 Materials and methods

### 2.1 Data collection

**Figure 1:**
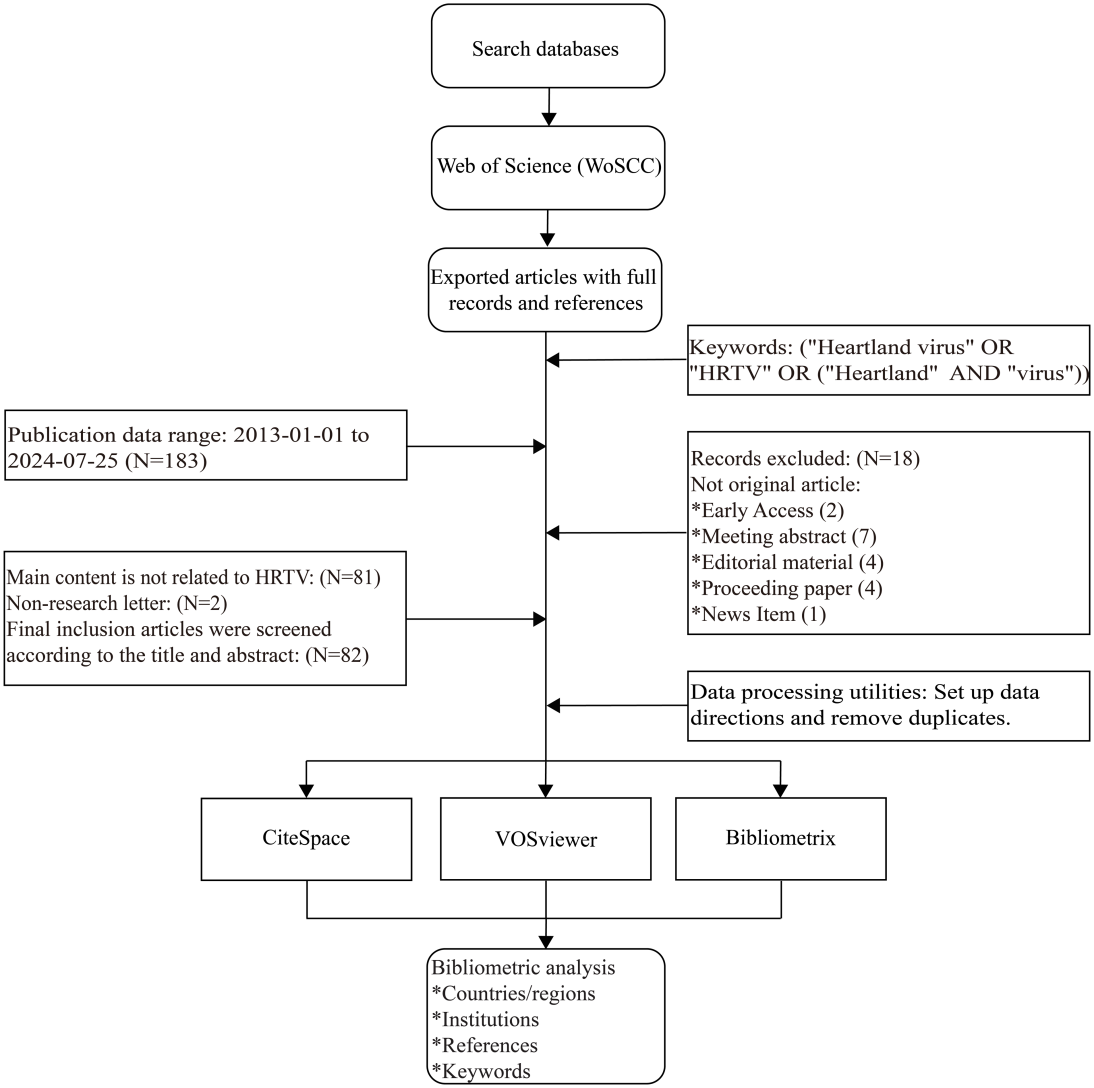
Process of data retrieval.

In this study, we chose WOSCC as the data source, which, as a highly respected database of high-quality digital literature resources, has been embraced by many researchers and is regarded as the database of choice for bibliometric analyses (Ding and Yang, 2022) Although the HRTV was first detected in Missouri in 2009, it only received further reporting and attention in 2012. During this time, although the virus was discovered, the associated scientific research had not yet developed sufficient scale and depth to reach the level of public publication. In our initial process of determining the start and end of the screening, our search keywords and strategy focused primarily on the literature in which the HRTV was a core research component; therefore, literature on the HRTV in 2012 was not directly identified as key literature, and thus the final determination was made that the screening would begin in 2013. We set the search formula as [“Heartland virus” OR “HRTV” OR (“Heartland” AND “virus”)] and then retrieved the literature from the WOSCC database for the period of January 1, 2013, to July 25, 2024. Document types included original research articles, reviews, and research letters in “English.” To ensure the timeliness and accuracy of the data, both researchers conducted an exhaustive literature search and screening of HRTV-related literature on the same day. A comprehensive database search was conducted based on title, abstract, and keywords as screening criteria. The full text of the literature was read to ensure its relevance to the purpose of the study when necessary. During the screening process, we need to pay special attention to well-documented literatures that contain important citation information and export these documents from the Web of Science platform to “TXT” format files for subsequent research and analysis. Figure 1 is a flowchart of the process of screening the literature focusing on HRTV as a core research component, demonstrating the process from the identification of keywords to the inclusion and exclusion of relevant literature, culminating in the analysis of multiple aspects of the included HRTV-related literature using three tools, CiteSpace, VOSviewer, and Bibliometrix for the write-up. Given that the data for this study were all sourced from a public database, there was no need to obtain additional ethics committee approval.

### 2.2 Data analysis and visualization

The beginnings of bibliometrics can date back to the dawn of the 20th century and it has subsequently played an important role in the field of literature analysis (Diem and Wolter, 2013). During the analysis phase, we can extract rich details covering authors, keywords, journal sources, country of affiliation, research organizations, and references. With this detailed data, we could gain insight and grasp a field’s history, current status, and future trends through bibliometric analysis (Abramo et al., 2011). In constructing the knowledge graph, we relied on two tools: CiteSpace software (version 6.2.4.24) and VOSviewer software (version 1.6.20). These two types of software complement each other and offer distinct advantages, significantly enhancing the efficiency of constructing knowledge graphs. CiteSpace is an interactive visualization tool based on set theory, focusing on data standardization. It uses clustering functions, combined with similarity measures and algorithms for knowledge units, aiming to deeply analyze and visualize the development lineage, research hotspots, and trends in HRTV and then gain insight into and predict the research frontier dynamics in the field (Synnestvedt et al., 2005). VOSviewer is probabilistic-based application software for scientific knowledge graphs, primarily used to create visual representations of knowledge graphs based on keyword co-occurrence, co-institution, and co-authorship. The software can vividly depict the development progress, coordination, and other relevant aspects of the HRTV field and is characterized by easy mapping and flexible operation. In addition, VOSviewer provides a variety of visualizations, such as network view, overlay view, and density view, offering a rich selection of data presentation options (van Eck and Waltman, 2010).

The bibliometric approach evaluates scientific research trends, impact, and quality by quantitatively analyzing academic literature. To enhance rigor and minimize limitations in our bibliometric study, we implemented the following strategies during the analysis: (1) A thorough literature screening was conducted to ensure data accuracy and reliability. (2) The characteristics and differences across various subject areas and types of literature were meticulously considered; for instance, we included only articles, reviews, and research letters to ensure the applicability and precision of our analysis results. (3) We gained a comprehensive understanding of the background of studies on HRTV diagnosis, transmission, and pathological processes, recognizing these as external factors that could influence the study outcomes.

VOSviewer is known for presenting a more comprehensive network structure, highlighting multiple relationships between nodes, which may result in a denser network but enable the identification of clear clusters and key nodes. Conversely, CiteSpace tends to focus on depicting critical paths and major clusters, offering more concise results. In our study, the key parameters of the CiteSpace software were consistently set, with the years per slice at 1. The sources for terms were concentrated on “Title,” “Author keywords,” and “Keywords Plus,” and the links’ scope was confined within slices. In the g-index, the scale factor k was set to 25. Despite formal differences in the outcomes presented by the two tools, there is significant overlap regarding major clusters and key nodes. These clusters highlight the distribution of various research topics or fields, while the key nodes represent influential literature or authors within the field. Analyzing these clusters and key nodes allows for an in-depth understanding of the research area.

In the data pre-processing stage, we used CiteSpace’s data processing utilities to de-duplicate and organize the included literature in WOSCC to ensure its accuracy. Moreover, we conducted a detailed standardization process for the overall collection of literature to guarantee its quality and uniformity. We created a fresh “TXT” file adhering to the “thesaurus_authors” format in the VOSviewer directory, where we consolidated synonyms. The implementation in the “TXT” file adhered to the following format: (1) Merging of synonymous terms, (2) Integration of subordinate units into higher-level entities, and (3) Consolidation of subordinate territories into their respective countries. Subsequently, we imported this file into the “VOSviewer thesaurus file” section on the clustering page of VOSviewer, achieving data reintegration. This data integration approach applies to countries, institutions, and references.

## 3 Results

### 3.1 Analysis of publications and citations

**Figure 2:**
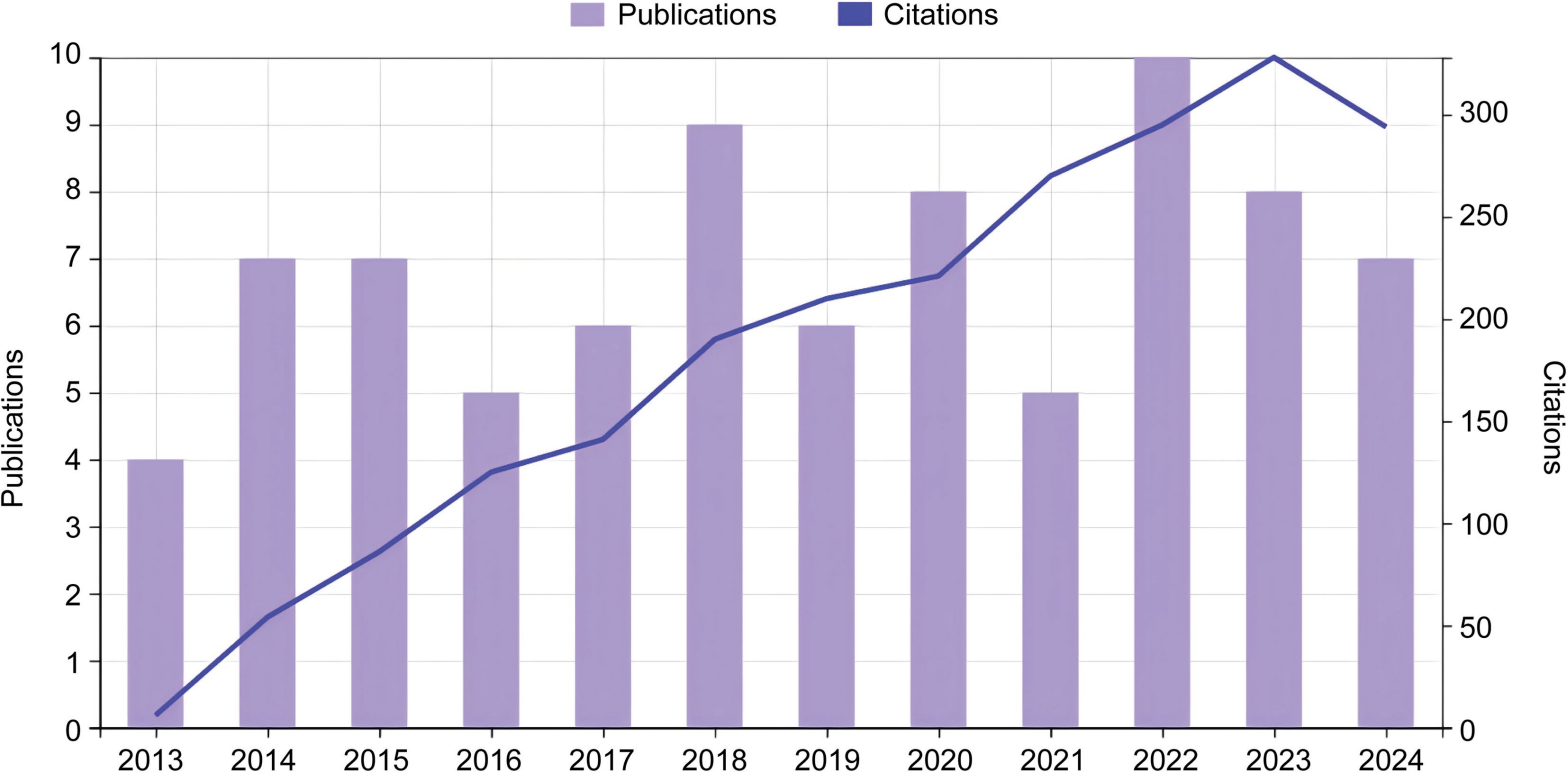
Trends of publications and citations from 2013 to 2024.

The search results of the WOSCC database were processed using CiteSpace, and a total of 82 relevant records were included, including 68 articles (82.93%), 13 reviews (15.85%), and one research letter (1.22%). Figure 2 shows the trend of publications and citations in the field of HRTV. From 2013 to 2024, the research in HRTV has maintained a steady and progressive growth trend. The average annual literature output has remained stable over the past decade, reflecting the broad interest and importance of the field in the research community. While some progress has been made in exploring HRTV, more in-depth research is still needed in pathogenesis, epidemiology, clinical manifestations, and therapeutic and prevention strategies. Since this study began in July 2024, the number of publications retrieved in that year showed a decreasing trend compared to previous years.

### 3.2 Analysis of countries/regions

A total of 17 countries and regions have published articles in the field of HRTV, and the top 5 countries with the most publications are shown in Table 1. Researchers in the United States published the largest number of papers in the HRTV field, 56 in total, followed by China and Japan, with 14 and 9 papers, respectively. The higher value of total link strength (TLS), as a measure of the tightness of connectivity between countries, reflects that the country is more actively engaged in cooperation with other countries. Table 1 shows that the United States and China are at the top of the list regarding excellence in international cooperation.

**Table 1:** Top 5 countries in a number of publications on HRTV.

Rank	Country	Publications	Total citations	Average citations	TLS	Centrality
1	The United States	56	1,514	27.04	7	0.21
2	China	14	326	23.29	7	0.48
3	Japan	9	193	21.44	6	0.06
4	Scotland	3	86	28.67	4	0.32
5	France	2	51	25.50	3	0.05

**Figure 3:**
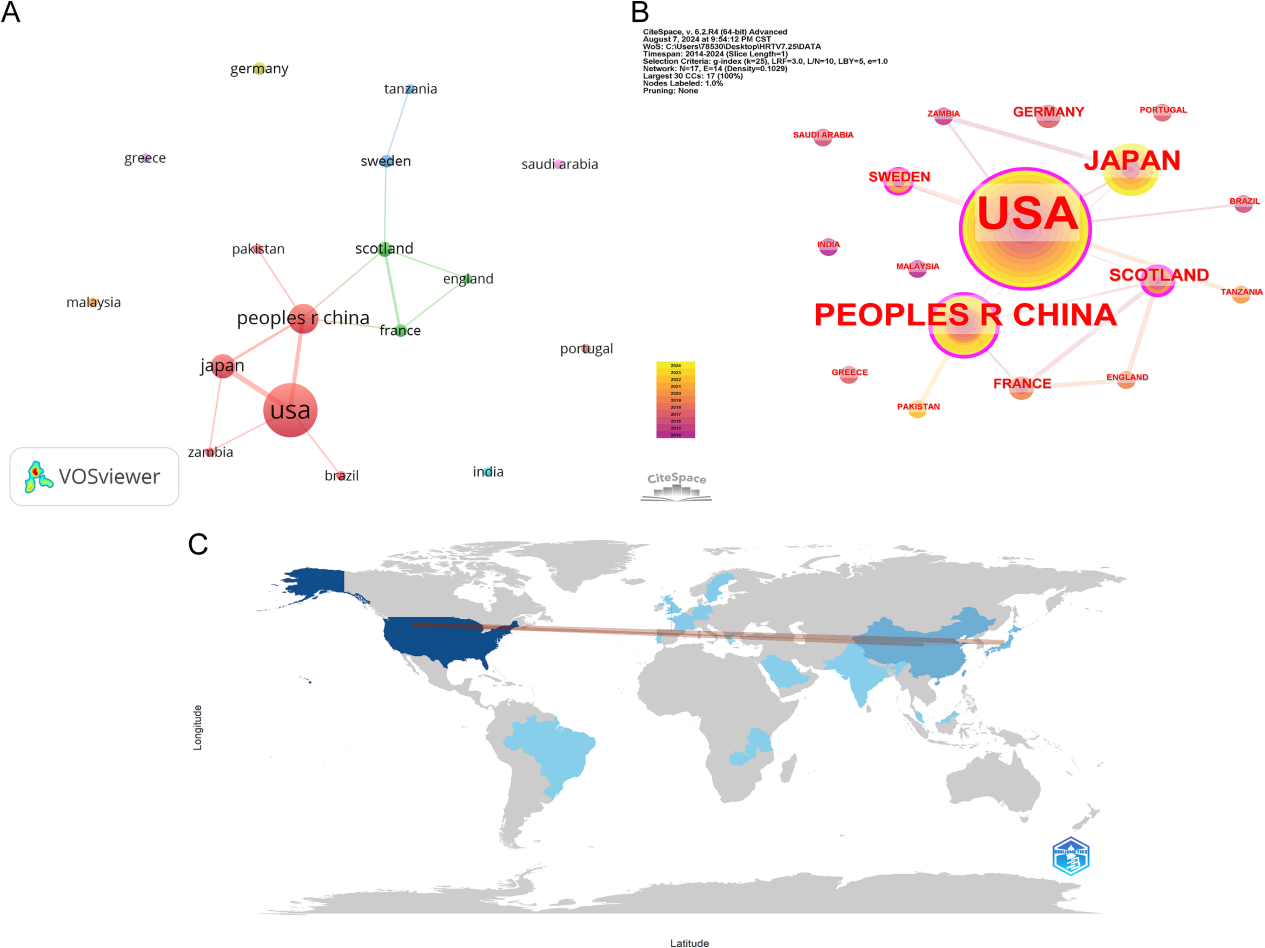
Visualization and analysis of the international collaboration networks in HRTV research. **(A)** Cooperation clustering map of countries. **(B)** Cooperation map of countries by citations. **(C)** Country collaboration map by scimago graphica.

We used VOSviewer software to visualize the countries and regions that have published at least one article. The results show that 17 countries and areas fulfill this condition, as presented in Figure 3A. The node size in the figure directly reflects the number of articles published in these countries and regions; the larger the node means, the more articles are published; while the connecting line between the nodes represents the strength of the cooperation association between these countries, the thicker the connecting line, the stronger the cooperation between the two countries. In Figure 3B, the size of the nodes is used to indicate the number of citations of articles from each country. The figure shows that the United States leads the total number of articles citations, followed by China, and Japan. Meanwhile, the color shades of the nodes indicate the articles’ publication year, with the darker color representing the earlier year. Summarizing the above analysis, the United States, China, and Japan are the most important contributors to the field of HRTV. The United States has many articles issued and the most frequent cooperation with China and Japan. However, there is an obvious imbalance in the distribution of issuing countries, with a few countries occupying a large number of research resources, forming a significant “top effect.” Academics pen a significant proportion of the papers in this domain from a limited number of nations. Figure 3C presents a map of the geographical distribution of international collaborations, with colored regions marking the core clusters of countries that have published significant papers in the field of HRTV. The thickness of the lines connecting these regions intuitively reflects the closeness of the collaborations between countries, with thicker lines implying a more significant number of papers published in collaboration between those two countries. In particular, within the field of HRTV research, the United States, China, and Japan all occupy a central position and have made key contributions to research in this area. Teamwork can effectively integrate resources, and international cooperation can introduce advanced technologies and concepts to enhance the research level and global influence.

### 3.3 Analysis of institutions

**Figure 4:**
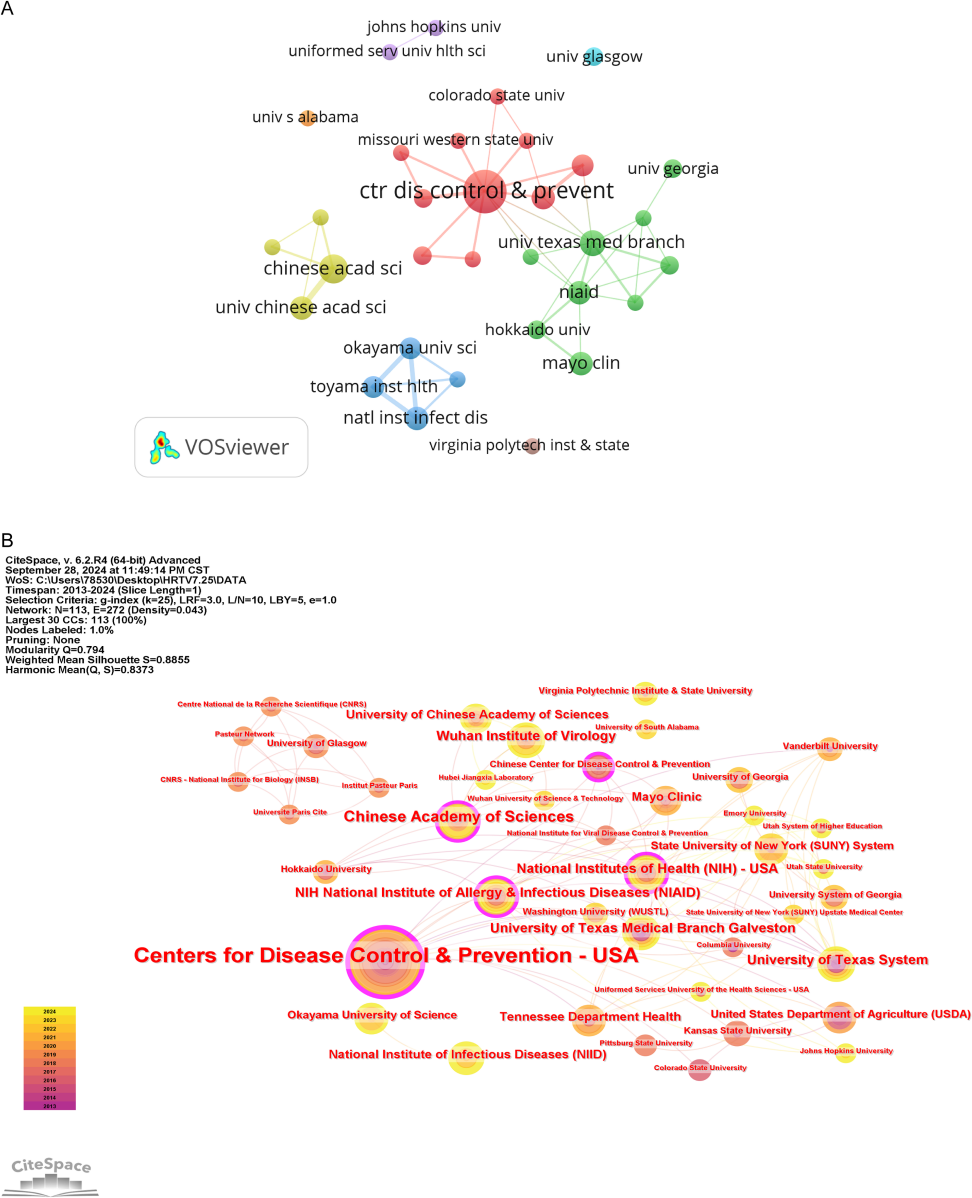
Visualization and analysis of the institutions in HRTV research. **(A)** Cooperation map of 32 institutions with the number of publications no less than 2 times. **(B)** Centrality cooperation map of institutions.

A total of 135 institutions published articles related to HRTV, and the top five institutions with the most publications, as shown in Table 2, were all from the United States and China, respectively, indicating that these two countries have the largest number of researchers in HRTV-related fields. The Centers for Disease Control and Prevention-USA had the highest number of publications (*n* = 20), followed by the Chinese Academy of Sciences (*n* = 8) and the University of Texas Medical Branch Galveston (*n* = 6). Setting the VOSviewer parameter to the minimum number of documents for an institution = 2 yielded 32 institutions. As shown in Figure 4A, the various colors symbolize different collaboration clusters, while the vast majority of collaboration clusters are confined to a single country. The connecting lines represent the strength of collaboration; the thicker the connecting lines represent the more collaborative documents between two institutions; the node size represents the number of documents; the larger the node, the more documents are sent. As illustrated in Figure 4B, we mapped the knowledge network of the collaborating institutions with the help of CiteSpace software. In this graph, the size of an organization’s node is directly related to its Betweenness Centrality (BC) in the knowledge network, with a larger node size indicating a higher BC value. This metric profoundly reveals the centrality of each node in the overall network architecture, thus effectively quantifying the extent of each institution’s contribution to the research results in the HRTV domain (Li and Zheng, 2022). Organizations such as the Centers for Disease Control and Prevention-USA (BC = 0.24), the Chinese Academy of Sciences (BC = 0.16), and the National Institutes of Health(NIH)-USA (BC = 0.15) play an important role in the collaborative network. Figures 4A,B illustrate that most publications in the field of HRTV originate from a select few organizations, such as ‘The Centers for Disease Control and Prevention-USA’ and ‘The Chinese Academy of Sciences,’ among others. Institutions with abundant resources are well-equipped to support research, and those prioritizing innovation tend to produce higher-quality and more influential literature. These institutions possess substantial academic foundations and significant professional advantages in HRTV research, thereby attracting numerous researchers to conduct studies in this field.

**Table 2:** Top 5 institutions in number of publications on HRTV.

Rank	Institution	Publications	Citations	Average citation/publication	Centrality
1	Centers for Disease Control and Prevention-USA	20	720	36.00	0.24
2	Chinese Academy of Sciences	8	168	21.00	0.16
3	University of Texas Medical Branch Galveston	6	202	33.67	0.04
4	National Institute of Allergy and Infectious Diseases	5	220	44.00	0.15
5	Mayo clinic	5	170	34.00	0.02

### 3.4 Analysis of authors

**Figure 5:**
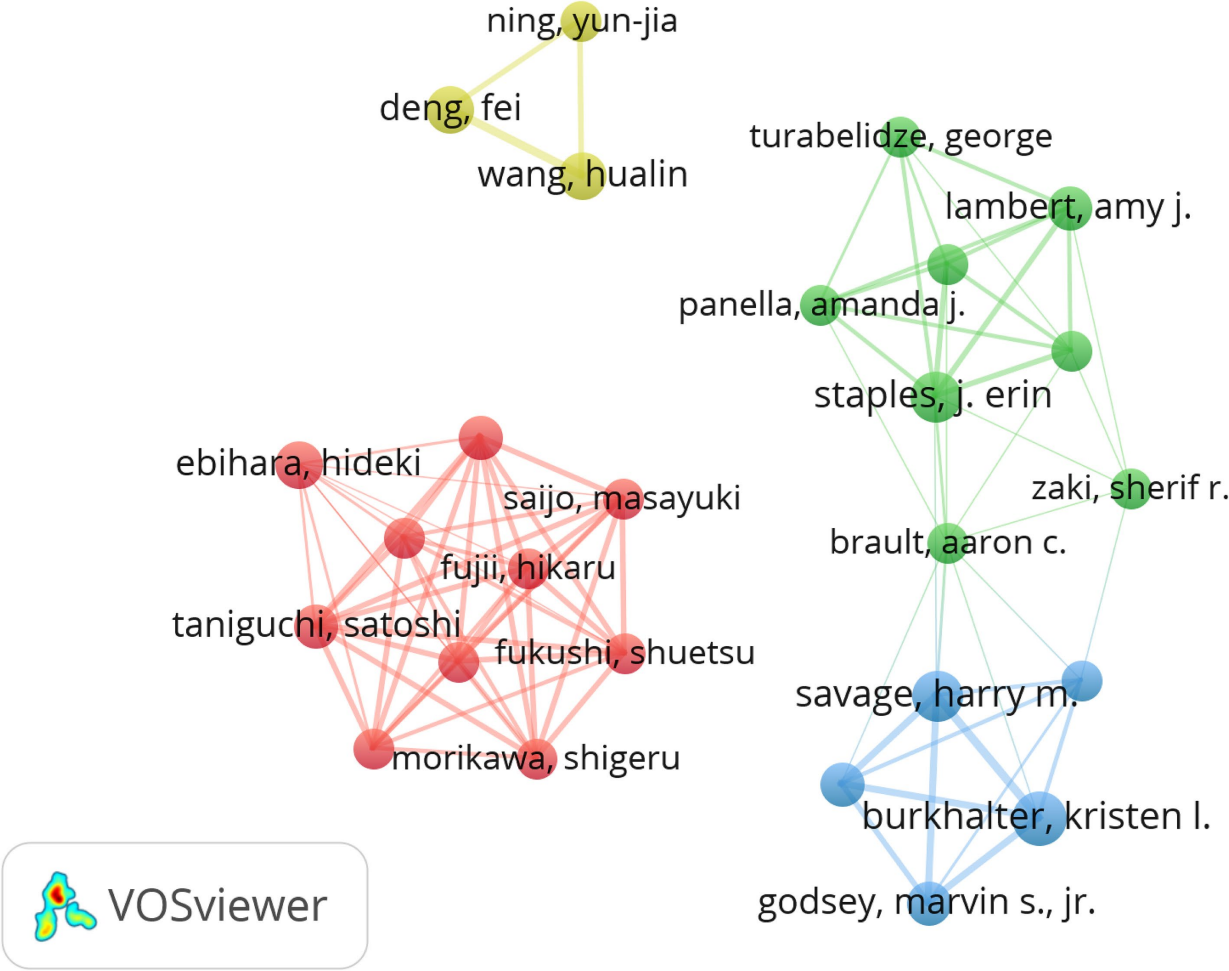
Cooperation map of 26 authors with the number of publications no less than 4 times.

The top three authors in terms of the number of published articles were Kristen L Burkhalter (*n* = 8), Savage HM (*n* = 7), and J. Erin Staples (*n* = 7) (Table 3). After analysis using CiteSpace software, we have included researchers who have published more than four articles related to HRTV in the co-author network mapping. The graph comprises 26 nodes and 87 connecting lines (Figure 5). This author collaboration graph is designed to reveal the most active and productive authors or co-authors and visualize their strong collaborative links. This tool provides researchers with information about influential research teams and facilitates their identification of potential partners, which in turn helps them build closer and more productive collaboration networks (Xia et al., 2022). As can be observed from the presentation in Figure 5, the 26 authors are divided into 4 different colored clusters. The connectivity between these clusters is sparse or almost non-existent, reflecting the very limited or practically non-existent cooperation between various clusters. Table 3 and Figure 5 indicate that a limited group of authors have both a high number of publications and a high citation count in the field of HRTV. The work of these highly influential authors tends to be more visible and frequently cited. Their research findings can significantly drive the development of the field. Although each of these authors possesses deep professional attainments in the field, they are fully capable of jointly producing more high-quality papers and scientific research results through enhanced cooperation and communication among themselves, thus promoting the further development of the field.

**Table 3:** Top 5 authors in HRTV field.

Rank	Author	Documents	Citations	Countries/Regions	Average citation/publication
1	Kristen L Burkhalter	8	373	The United States	46.63
2	Savage HM	7	417	The United States	59.57
3	J. Erin Staples	7	243	The United States	34.71
4	Hualin Wang	6	152	China	25.33
5	Hideki Ebihara	6	172	Japan	28.67

### 3.5 Analysis of references

**Figure 6:**
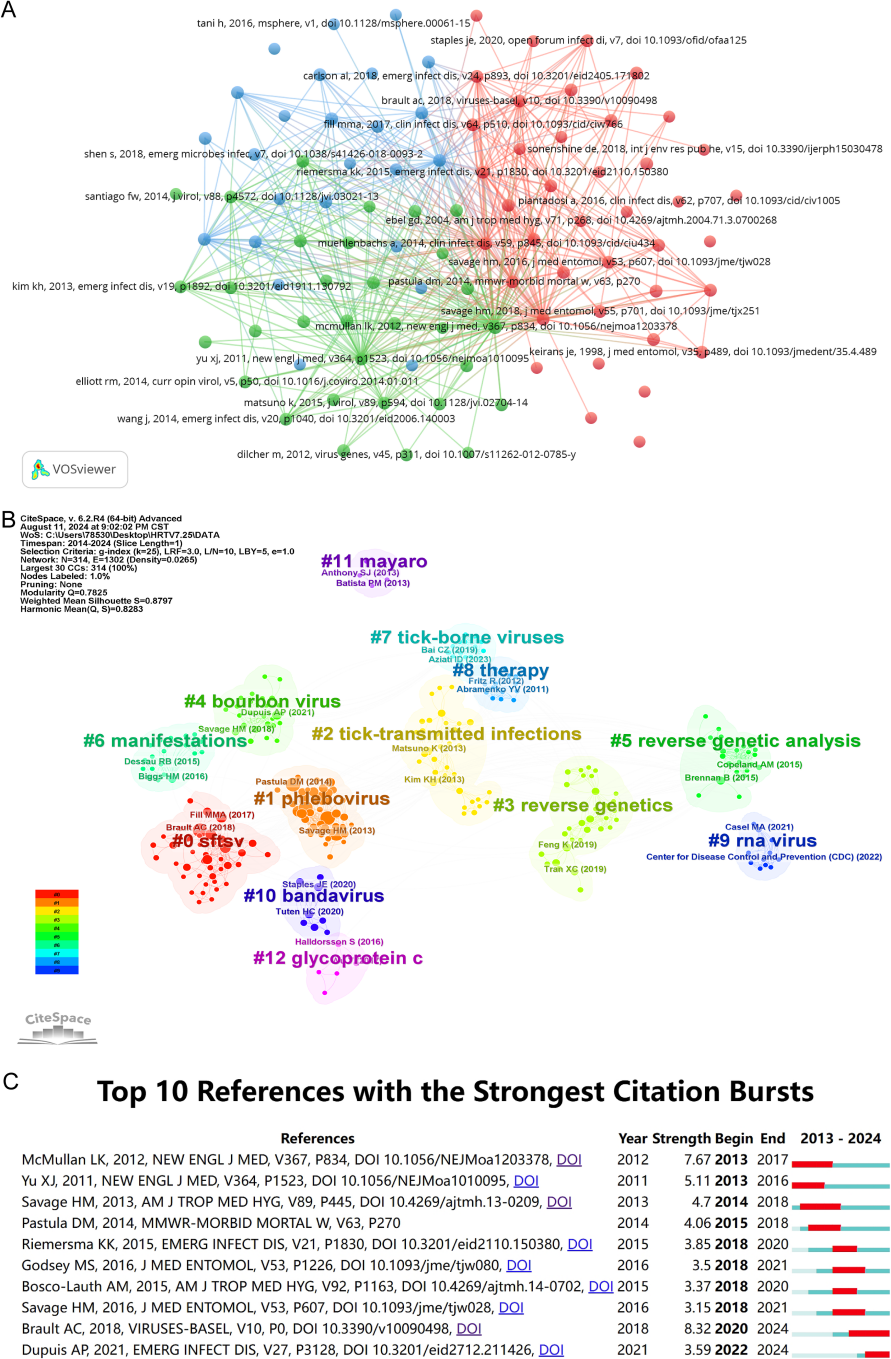
Visualization and analysis of the references in HRTV research. **(A)** Distribution of 97 references with a frequency of no less than 5 times. **(B)** References co-citation clustering network. **(C)** Top 10 references with the strongest citation bursts.

The 82 articles included in this study cited a total of 2,236 references. As shown in Table 4, the article by McMullan et al. (2012) featured prominently in terms of the number of co-citations, which strongly suggests that this paper’s research value and impact in the field of HRTV are extremely prominent. By applying VOSviewer for co-cited literature analysis, we set a minimum value of 5 for co-cited literature and found that 97 articles reached this threshold. As presented in Figure 6A, the figure demonstrates a reference network consisting of three different clusters. Thirteen major sub-themes related to HRTV were identified using the reference clustering feature of CiteSpace, as shown in Figure 6B. Based on the analyzed data, we can see that the Modularity Q is as high as 0.7825 (>0.3), and the weighted mean Silhouette S reaches 0.8797 (>0.7), both of which are high. The clustering structure is clear and significant in the co-cited cluster analysis, and the clustering results are highly convincing. The Modularity Q and the weighted mean Silhouette S demonstrate excellent clustering results. Citation explosion depicts the phenomenon of certain keywords suddenly emerging or the citation frequency climbing dramatically within a short period, which becomes a key indicator and a significant manifestation of the evolution of research hotspots over time. As shown in Figure 6C, this figure depicts the top 10 documents with the strongest citation explosion. “Heartland Virus Epidemiology, Vector Association, and Disease Potential” was the strongest citation outbreak in 2018, with an intensity of 8.32, reflecting the importance of this article to the HRTV field. This literature provides a more detailed assessment of the natural transmission cycle of HRTV, and it utilizes the dual strategy of serological surveys and enhanced HRTV disease surveillance to provide in-depth insights into the transmission mechanisms and dynamics of HRTV, thereby more accurately assessing the potential risk it poses to human health. These efforts have provided a valuable scientific basis for the development of effective HRTV prevention and control strategies and have made a notable contribution in this area.

**Table 4:** Top 10 references in HRTV field.

Rank	Author	Co-citations	Year	Journals	DOI
1	McMullan LK	66	2012	New Engl J Med	10.1056/NEJMoa1203378
2	Savage HM	48	2013	Am J Trop Med Hyg	10.4269/ajtmh.13-0209
3	Yu XJ	40	2011	New Engl J Med	10.1056/NEJMoa1010095
4	Muehlenbachs	35	2014	Clin Infect Dis	10.1093/cid/ciu434
5	Pastula DM	34	2014	Mmwr-morbid Mortal W	10.3390/v10090498
6	Bosco-Lauth AM	27	2015	Am J Trop Med Hyg	10.4269/ajtmh.14-0702
7	Riemersma KK	26	2015	Emerg Infect Dis	10.3201/eid2110.150380
8	Brault AC	24	2018	Viruses-basel	10.3390/v10090498
9	Bosco-Lauth AM	22	2016	Am J Trop Med Hyg	10.4269/ajtmh.14-0702
10	Fill MMA	18	2017	Clin Infect Dis	10.1093/cid/ciw766

### 3.6 Analysis of journals

**Figure 7:**
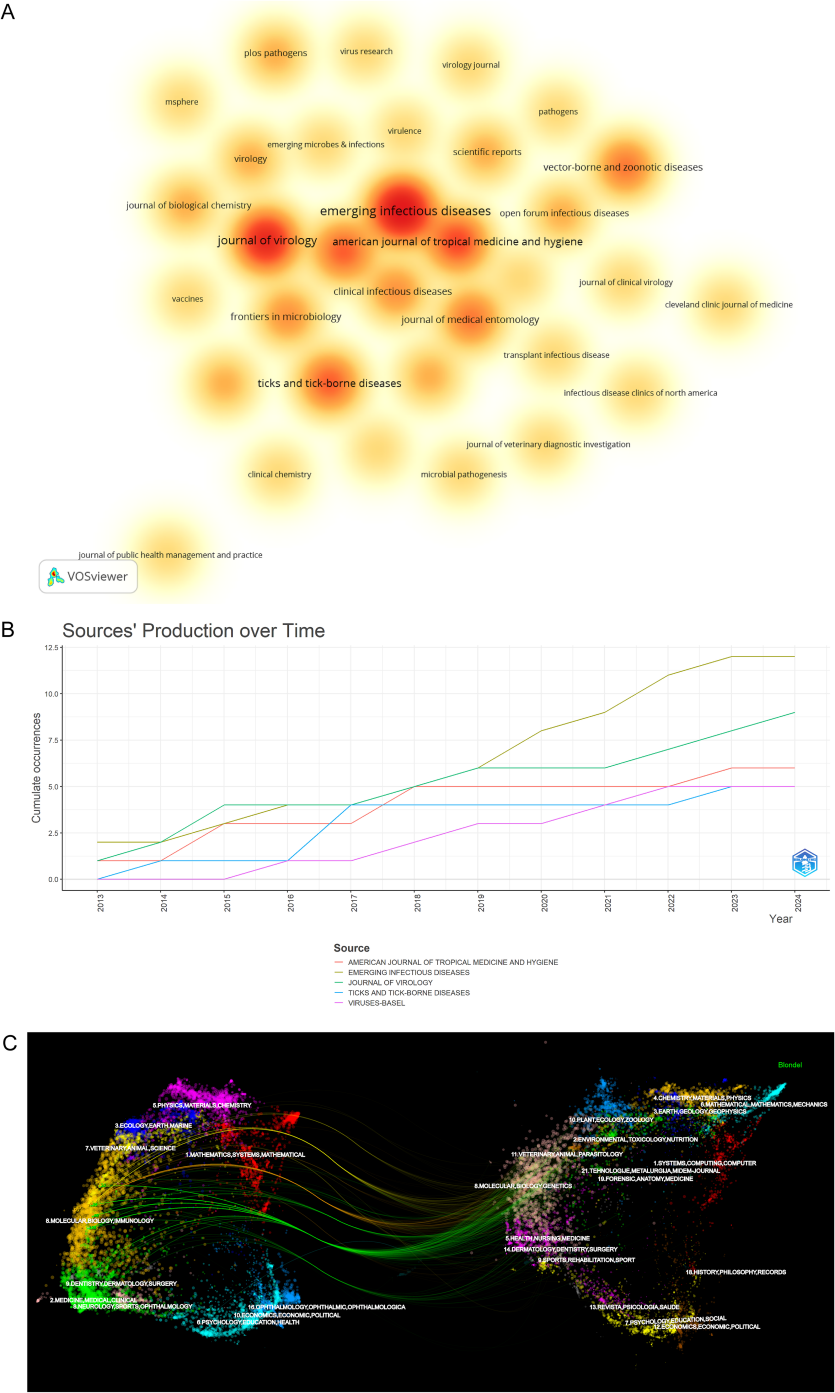
Visualization and analysis of the top journals in HRTV research. **(A)** Density visualization of journals in HRTV field. **(B)** Sources’ production over time from 2013 to 2024 in HRTV field. **(C)** Dual-map of journals on HRTV research.

The 82 articles included in this study were published in 33 journals. As shown in Table 5, among the top 10 published journals in HRTV, the top 3 journals were Emerging Infectious Diseases (IF = 7.2), Journal of Virology (IF = 4.0), and American Journal of Tropical Medicine and Hygiene (IF = 1.9), with 12, 9, and 6 articles published, respectively. Journal of Virology (IF = 4.0), Emerging Infectious Diseases (IF = 7.2), and American Journal of Tropical Medicine and Hygiene (IF = 1.9) were the top 3 cited journals. Figure 7A presents an overview of the density visualization of journals publishing literature in the field of HRTV, where the shade of the colors directly maps the amount of literature on HRTV published by the journal. Specifically, darker colors indicate that the journal is more prolific in publishing literature in this field. The three journals that contributed the most to the field were the Emerging Infectious Diseases, the Journal of Virology, and the American Journal of Tropical Medicine and Hygiene. Figure 7B shows the trends in publication volume related to HRTV in five different journals from 2013 to 2024. Emerging Infectious Diseases and the Journal of Virology have been important in the HRTV-related field for decades. Figure 7C is a biplot overlay of HRTV research journals. The cluster positioned on the left signifies the group of journals engaging in citations, whereas the cluster situated on the right encapsulates the collection of journals that are being cited. The citing journals are mainly concentrated in one field: Molecular, Biology, Immunology. The cited journals are mainly concentrated in three fields: (1) Molecular, Biology, Genetics; (2) Health, Nursing, Medicine; (3) Veterinary, Animal, Parasitology. Figures 7A,B show that most articles in the field of HRTV are concentrated in specific journals, such as “Emerging Infectious Diseases” and the “Journal of Virology.” These journals are noted for their high professionalism and authority in this area, ensuring the quality and academic rigor of the articles they publish. Consequently, they are often the preferred choice for authors in this field. Furthermore, HRTV research is interdisciplinary, encompassing medical, biological, and chemical aspects, and its advancement relies on the development of related disciplines and multidisciplinary collaboration. Compared to similar viruses like SFTSV, (Zhang and Zhang, 2024) HRTV research is still in its nascent stages, with research hotspots needing to transition from exploring basic technology to applying cutting-edge technology.

**Table 5:** Top 10 journals in number of publications and citations in HRTV field.

Rank	Publication journal	Documents	Citations	IF*	Cited journal	Co-citations	IF*
1	Emerging Infectious Diseases	12	252	7.2	Journal of Virology	374	4.0
2	Journal of Virology	9	288	4.0	Emerging Infectious Diseases	286	7.2
3	American Journal of Tropical Medicine and Hygiene	6	245	1.9	American Journal of Tropical Medicine and Hygiene	191	1.9
4	Viruses-Basel	5	207	3.8	New England Journal of Medicine	137	96.2
5	Ticks and Tick-Borne Diseases	5	111	3.1	Clinical Infectious Diseases	122	8.2
6	Journal of Medical Entomology	4	140	2.1	Journal of Medical Entomology	107	2.1
7	Vector-Borne and Zoonotic diseases	4	86	1.8	Virology	82	2.8
8	Clinical Infectious Diseases	3	110	8.2	Viruses-Basel	76	3.8
9	Frontiers in Microbiology	3	70	4.0	Journal of General Virology	70	3.6
10	Journal of biological chemistry	2	66	4.0	PLoS One	66	2.9

IF*, impact factor.

### 3.7 Analysis of keywords in HRTV research

**Figure 8:**
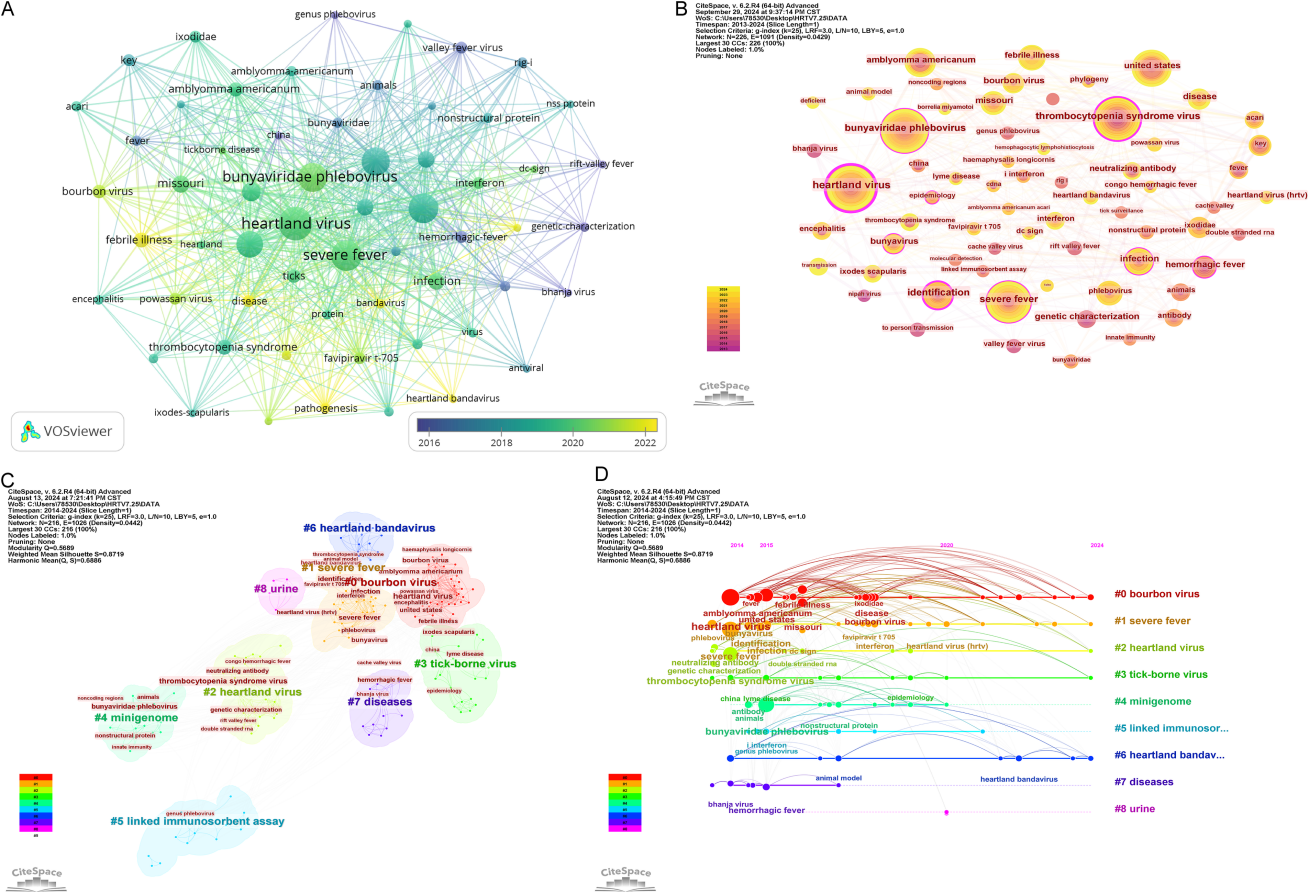
Visualization and analysis of the keywords in HRTV research. **(A)** Distribution of 59 keywords with an average publication of no less than 3 times. **(B)** Co-occurrence of keywords. **(C)** Keywords clustering network. **(D)** Timeline view of keywords cluster.

The keyword refinement accurately summarizes a paper’s core content and essence, and the keyword co-occurrence analysis can be used to identify the research hotspots in HRTV-related fields. The most common keyword is “Heartland virus” (Table 6). The keywords that exhibited the greatest centrality were “Heartland virus” and “identification,” while other keywords with higher centrality included “thrombocytopenia syndrome virus,” “hemorrhagic fever,” and “severe fever” (Table 7). Figure 8A shows the overlay visualization of the keywords using VOSviewer. The node size represents the average frequency of the keyword per year; the larger the node, the higher the average annual frequency of the keyword. The four colors, from dark to light, represent the time order of the keywords from first to last; the darker the color, the earlier the year the keyword appears. The darker the color, the earlier the year in which the keyword appeared. It can be seen that the keywords with the highest average annual frequency in recent years are “Heartland virus” and “severe fever.” Figure 8B shows the keyword co-occurrence map produced by CiteSpace; each node consists of different colored annual rings, where the larger the node means, the higher the total frequency of the keyword, and the shades and thicknesses of the annual rings represent the early and late time of the occurrence and the frequency of the corresponding year, respectively. From the figure, it can be seen that “Heartland virus” and “severe fever” are the focus of HRTV research. Cluster analysis using CiteSpace keywords resulted in 9 clusters with a Modularity Q of 0.5689 and a weighted mean Silhouette S value of 0.8719, which resulted in a significant cluster structure and credible clustering results (Figure 8C). In the timeline visualization (Figure 8D), the keywords of the nine clusters were depicted along the horizontal timeline, which depicted the research pulse and developmental dynamics in the field of HRTV as well as the diagnostic methods, risk factors, pathogen transmission routes related to HRTV, and the potential interconnections among them between 2013 and 2024.

**Table 6:** Top 10 keywords by frequency in HRTV field.

Rank	Keywords	Frequency
1	Heartland virus	32
2	Severe fever	28
3	Thrombocytopenia syndrome virus	26
4	Bunyaviridae phlebovirus	25
5	Phlebovirus	24
6	The United States	22
7	Transmission	13
8	Missouri	11
9	Infection	10
10	Identification	10

**Table 7:** Top 5 keywords by centrality in HRTV field.

Rank	Keywords	Centrality
1	Heartland virus	0.48
2	Identification	0.28
3	Thrombocytopenia syndrome virus	0.24
4	Hemorrhagic fever	0.17
5	Severe fever	0.16
6	Bunyavirus	0.16
7	Bunyaviridae phlebovirus	0.13
8	Infection	0.13
9	Epidemiology	0.10
10	Disease	0.08

## 4 Discussion

HRTV disease, first identified 15 years ago, is a tick-borne disease contracted by the HRTV through the tick bite of the Lone Star Tick (*Amblyomma americanum*) and potentially other tick species (McMullan et al., 2012). HRTV is an emerging and not yet fully understood virus that can cause severe sequelae after infection (Dembek et al., 2024). Typical clinical symptoms of HRTV infection include fever, generalized weakness, headache, nausea, diarrhea, and muscle pain. Laboratory tests reveal features including decreased white blood cell and platelet counts, elevated liver enzyme levels, and the potential for a rapidly fatal, widely disseminated infection accompanied by multi-system organ failure, even in the absence of severe complications (Muehlenbachs et al., 2014; Pastula et al., 2014; Fill et al., 2017). HRTV exhibits a strong genetic relatedness to SFTSV and exhibits similar pathology in China, Korea, and Japan (Brault et al., 2018). The spread of HRTV has occurred primarily in the southern and midwestern states of the United States, in addition to its documented presence outside the south and midwestern United States; however, with habitat expansion and tick range expansion due to climate warming, the actual reach of this infection may be much broader than we currently understand (Dembek et al., 2024). Given the importance of exploring the transmission of HRTV and the mechanisms that cause the disease, an urgent need is to develop an animal model that meets several conditions, such as mimicking natural transmission and exhibiting symptoms and manifestations similar to those of known human cases. Efforts have been made to develop a suitable animal model, but these attempts are still limited because immunocompetent animals often do not fully mimic the symptoms of human disease. After several experimental studies in which several different vertebrates (including hamsters, goats, chickens, raccoons, rabbits, interferon alpha/beta/gamma receptor-deficient [Ag129] mice and C57BL/6 mice) have been inoculated with HRTV viruses, the A129 mouse has been characterized as the best small animal model for mimicking the pathogenesis of clinical HRTV in humans and has provided a powerful tool for research in HRTV-related areas (Bosco-Lauth et al., 2016; Brault et al., 2018; Reynolds et al., 2023).

HRTV belongs to the family *Phenuiviridae* and the genus *Bandavirus*, and its genome structure consists of multiple-segmented single-stranded negative-sense RNAs. These RNA segments include small (S), medium (M), and large (L) sizes, which each carry different genetic information (Bosco-Lauth et al., 2016; Kimura et al., 2021). Among them, the coding sequences of nucleocapsid (N) proteins and nonstructural (NSs) proteins are located on the S fragment. The M fragment is responsible for encoding the structural glycoproteins Gn and Gc, both viral surface glycoproteins, which are essential during the initial stages of viral infection. The direct transcription process of the virus depends on the RNA-dependent RNA polymerase encoded on the L fragment (Swei et al., 2013). Researchers successfully resolved the crystal structure of HRTV Gc in the post-fusion conformation. The Gc extracellular domain of HRTV showed a 71% sequence similarity to SFTSV Gc. The two Gc proteins showed a high degree of overall structural identity. This finding provides a new perspective for the comprehensive comparative study of class II membrane fusion proteins in Bunyavirus and valuable reference information for treating these pathogens that affect humans (Zhu et al., 2018).

The main challenge in treating HRTV infections has been the need for more effective antiviral drugs. Currently, no confirmed antiviral treatments are available to address HRTV infections; therefore, the acute phase management is focused on supportive care. Antipyretics and analgesics are effective in reducing fever and pain. Further treatment, including ventilator support, vasopressor medications, blood transfusions, or dialysis, may be necessary for patients with severe disease (Brault et al., 2018; Suleman et al., 2022). Among the potential therapies for HRTV infection that have not yet been practically applied are tanshinone I and IIa, favipiravir, the NF-κB inhibitor SC75741, and anidulafungin. Based on the development studies for SFTSV and HRTV vaccines, it is predicted that these vaccines may effectively prevent HRTV infections. (Dembek et al., 2024) Successful primary prevention and control measures for tick-borne viral infections encompass vaccination, personal protective practices, landscape maintenance, and wildlife oversight (Diaz, 2020).

SFTSV and the emerging virus HRTV cause serious human diseases in the Far East and the US region, respectively. Manipulating these viruses in a laboratory setting through reverse genetics techniques will greatly advance the in-depth study and understanding of these emerging pathogens, providing strong support for developing antiviral drugs and creating protective vaccines (Brennan et al., 2015). In the treatment of cases involving SFTSV, although a variety of medications, including steroids and ribavirin, have been used with the expectation of reducing morbidity and mortality from the disease, these therapeutic attempts have not yielded the desired results, and these therapies have proven to be ineffective (Liu et al., 2014; Suleman et al., 2022). The febrile symptoms triggered by SFTSV and HRTV due to an aberrant host inflammatory response lead to a disease resembling human viral hemorrhagic fever, which is key to its pathogenesis. Favipiravir is a nucleoside analog that has demonstrated significant therapeutic efficacy *in vitro* experiments and a rodent model of rodents with defective type I IFN signaling in the presence of SFTSV or HRTV infection (Westover et al., 2017). Researchers constructed an M-segment microgenomic system based on SFTSV and HRTV to screen potential compounds capable of inhibiting viral RNA synthesis. Through this system, the NF-κB inhibitor SC75741 was verified to reduce viral RNA synthesis in SFTSV and HRTV significantly, thus providing a novel approach to screen anti-inflammatory compounds and greatly facilitating the rapid evaluation process of therapeutic drug candidates (Mendoza et al., 2021). The life cycle of HRTV and its interaction with host cells is largely speculative and primarily inferred from the knowledge of other Bunyaviruses. To gain a better understanding of the life cycle and the host factors that regulate viral infection, there is a need to intensify research into HRTV-host interactions. Meanwhile, the pathogenesis and virulence of several other Bunyaviruses, especially SFTSV, have been studied in greater detail. Comparative studies, based on existing knowledge of other Bunyaviruses, are crucial to uncover the similarities and differences with HRTV and to quickly comprehend its virological properties. Furthermore, the development of vaccines and drugs against HRTV infection is an urgent priority. Several promising SFTSV vaccine candidates and anti-SFTSV drugs have been reported, and these strategies could be adapted to evaluate their inhibitory effects on HRTV.

In studies of HRTV infection, several host factors have been found to have a moderating effect on viral infection. Patients’ physical condition is more influential, and their risk of death is significantly higher when they are accompanied by comorbidities such as diabetes mellitus, chronic viral hepatitis, central nervous system complications, and bacterial or fungal co-infections. In addition, it has been shown that tick salivary gland extract has an enhancing effect on HRTV infection and its pathogenicity in A129 mice. Thus, a more in-depth investigation into the mechanisms of the interactions among pathogens, hosts, and vectors is necessary (Muehlenbachs et al., 2014). Future studies using the A129 mouse model will deepen our knowledge of HRTV transmission and pathogenesis and hopefully reveal key tick saliva components that influence the disease process. Resolving these specific components could provide potential targets for vaccine development, which could help stop disease transmission or prevent tick bites by blocking their feeding behavior, further reducing the risk of disease transmission (Reynolds et al., 2023). Innate immunity is also important, with IFN receptor-deficient and STAT1/STAT2 knockout animals showing higher susceptibility than immunocompetent individuals (Kimura et al., 2021). Age and gender also play an important role in the modulatory effect of viral infection, with most patients infected with HRTV being elderly males. This feature is consistent with the results of SFTSV infection (Zhao et al., 2021). Autophagic factors should not be neglected; HRTV may trigger the autophagic process and enhance its replication capacity by regulating autophagy (Sun et al., 2021). With the growing understanding of HRTV disease, we can collect more data on its higher severity clinical features and its associated risk factors (Carlson et al., 2018). The intricate interactions between HRTV and various host factors are highly likely to significantly accelerate the pathogenesis of the virus and drive its spread. Such interactions play a key role in the life cycle of HRTV and significantly impact the virus’s survival, replication, and eventual spread from the host to other hosts.

Visual analysis of bibliometrics can review and summarize research results and provide insights and predictions about future research frontiers and challenges in the academic field. However, we must still be aware of the limitations when using tools like CiteSpace, VOSviewer, and Bibliometrix for bibliometric analysis. The data we searched were mainly based on English publications in the WOSCC database. Even though this database enjoys a high degree of authority in academia and is generally recognized as a high-quality bibliographic resource, some documents still need to be included, which may lead to an underestimation of citation counts and thus introduce small biases in our findings. Second, this study’s relatively long period and its focus on quantitative analysis may have overlooked some important details to a certain extent. Finally, regarding the use of citation data, the most recent year’s data is unstable and dynamic, changing over time, which can affect the robustness of the analysis to some extent. However, with the continuous innovation in science, technology, and medicine, the visual analysis of bibliometrics will continue to improve in accuracy and provide more solid and reliable support for academic research. The uncovered research hotspots and emerging trends in HRTV will offer researchers ample inspiration and fresh avenues for investigation.

The visualization analysis in this study requires improvement compared to bibliometric analyses in related fields (Zhang and Zhang, 2024). First, the theme of this study is not as prominent as those in similar analyses. Given the limited amount of literature on HRTV (fewer than 100 articles in total) and the possibility of confusing some articles with those on similar viruses like SFTSV, there is a risk of thematic shifts in the visual analysis results. This is one of the key factors affecting the study’s outcomes. Second, since HRTV has been reported in the literature for only 10 years, the period of study is relatively short, and the research lacks depth. Consequently, the visualization results are somewhat limited.

Using bibliometric analysis and data visualization, we review and integrate the research findings in the field of HRTV over the past decade and look forward to future research directions and possible challenges. Academic interest in HRTV continues to grow, and the annual publication volume of related literature maintains a stable trend. The United States is the leader in HRTV research, and the cooperation and exchange between countries and the publication of research results have shown an increasing trend. This trend highlights the importance of international collaboration and knowledge exchange in producing high-quality research results. Current vaccine development and immune response research is dedicated to deepening the understanding of viral disease progression in order to optimize clinical outcomes. Currently, HRTV research focuses on the pathogenic mechanisms and the associated immune responses, reflecting the urgent need for efficient treatment and prevention strategies.

## Data Availability

The original contributions presented in the study are included in the article/supplementary material, further inquiries can be directed to the corresponding author.
